# Analysis of Benzodiazepine Prescription Practices in Elderly Appalachians with Dementia via the Appalachian Informatics Platform: Longitudinal Study

**DOI:** 10.2196/18389

**Published:** 2020-08-04

**Authors:** Niharika Bhardwaj, Alfred A Cecchetti, Usha Murughiyan, Shirley Neitch

**Affiliations:** 1 Department of Clinical and Translational Science Joan C Edwards School of Medicine Marshall University Huntington, WV United States; 2 Department of Internal Medicine Joan C Edwards School of Medicine Marshall University Huntington, WV United States

**Keywords:** dementia, Alzheimer disease, benzodiazepines, Appalachia, geriatrics, informatics platform, interactive visualization, eHealth, clinical data

## Abstract

**Background:**

Caring for the growing dementia population with complex health care needs in West Virginia has been challenging due to its large, sizably rural-dwelling geriatric population and limited resource availability.

**Objective:**

This paper aims to illustrate the application of an informatics platform to drive dementia research and quality care through a preliminary study of benzodiazepine (BZD) prescription patterns and its effects on health care use by geriatric patients.

**Methods:**

The Maier Institute Data Mart, which contains clinical and billing data on patients aged 65 years and older (N=98,970) seen within our clinics and hospital, was created. Relevant variables were analyzed to identify BZD prescription patterns and calculate related charges and emergency department (ED) use.

**Results:**

Nearly one-third (4346/13,910, 31.24%) of patients with dementia received at least one BZD prescription, 20% more than those without dementia. More women than men received at least one BZD prescription. On average, patients with dementia and at least one BZD prescription sustained higher charges and visited the ED more often than those without one.

**Conclusions:**

The Appalachian Informatics Platform has the potential to enhance dementia care and research through a deeper understanding of dementia, data enrichment, risk identification, and care gap analysis.

## Introduction

Dementia is the fifth leading cause of death among people older than 65 in the United States [1]. The prevalence of dementia has been escalating, especially in West Virginia, a state with one of the highest percentages of older adults in its population [2]. Not only that, but more than half (52.5%) of these older adults also reside in rural areas [3]. As of early 2019, an estimated 38,000 people with Alzheimer’s disease (AD) were living in West Virginia, and this number is expected to increase to 44,000 by 2025 [4]. Although age is the greatest risk factor for AD, comorbidities such as stroke, cardiovascular disease, smoking, high cholesterol, obesity, poor nutrition, physical inactivity, and diabetes also contribute to the disease burden in AD [5]. According to the Centers for Disease Control and Prevention’s Behavioral Risk Factor Surveillance System, West Virginia has been ranked among the worst of the 50 states and District of Columbia in the prevalence of smoking, diabetes, hypertension, and obesity [6]. Associated contributory factors to dementia, such as excessive prescription of medications (eg, benzodiazepines [BZD]), poor rates of health screening, and high illiteracy, have also been found to be highly prevalent in West Virginia and the rest of the Appalachia [7-9]. Thus, caring for patients with dementia is challenging, especially in Appalachia, because of the complexities that arise due to the increased burden of aforementioned comorbidities and contributory factors [10]. Moreover, 42% of the Appalachian population resides in remote and rural settings, limiting access to health care [11,12].

Technological advancements over the past few years have only added to the challenges by leading to the production of massive amounts of data, known as big data, originating from a wide variety of disparate sources, such as electronic health records (EHRs), specialized registries, smart home health devices, genomic data, etc [13-16]. In order to transform this siloed data into actionable knowledge to further dementia research and care, it is vital to connect them and create a longitudinal record across the care continuum. This can be achieved through the application of numerous current and emerging big data approaches for data storage, management, analytics, and mining [17]. These techniques offer benefits such as data quality, data structure, data accessibility, quality improvement, population management and health, early detection of disease, improved decision making, and cost reduction. However, they pose some challenges concerning security, infrastructure, ethics, and scientific evidence and theory that still need to be overcome [18-26].

The Appalachian Clinical and Translational Science Institute (ACTSI) at Marshall University Joan C Edwards School of Medicine (MU/JCESOM) has recently established the Maier Institute for Excellence in Therapeutics for Elders with Dementia, which aims to enhance patient care and advance research in AD and other dementias in the Appalachian elderly population. The focus of Maier Institute is on strategic approaches that will identify existing gaps and improve the quality of care for patients with dementia. The Maier Institute is dedicated to ensuring that every person with dementia receives optimal treatment through our discovery of new knowledge and dissemination of information regarding appropriate therapeutics.

This paper describes one of the Maier Institute's approaches to improving therapeutics for this very vulnerable population using the Appalachian Informatics Platform, which will be very valuable going forward in our pursuit of the mission of Maier Institute. As a foundation for all future studies, the ACTSI Maier Institute Data Mart was built using the Clinical Data Warehouse (CDW), which will serve as a source of consolidated information across the care continuum for our geriatric population. Using data from this data mart, a pilot exploratory study was conducted.

Given the current climate of crisis in the use of controlled and addictive drugs and the potential for abuse of BZDs, we studied the prescribing patterns of the BZD class of medications within our patient population. While very useful when properly prescribed, BZDs can be harmful to elderly persons otherwise. Thus, the goal of this paper was to provide a better understanding of their use, which is critical to good clinical care, through the use of the Appalachian Informatics Platform, thereby demonstrating the value of this platform in driving dementia research.

## Methods

**Figure 1:**
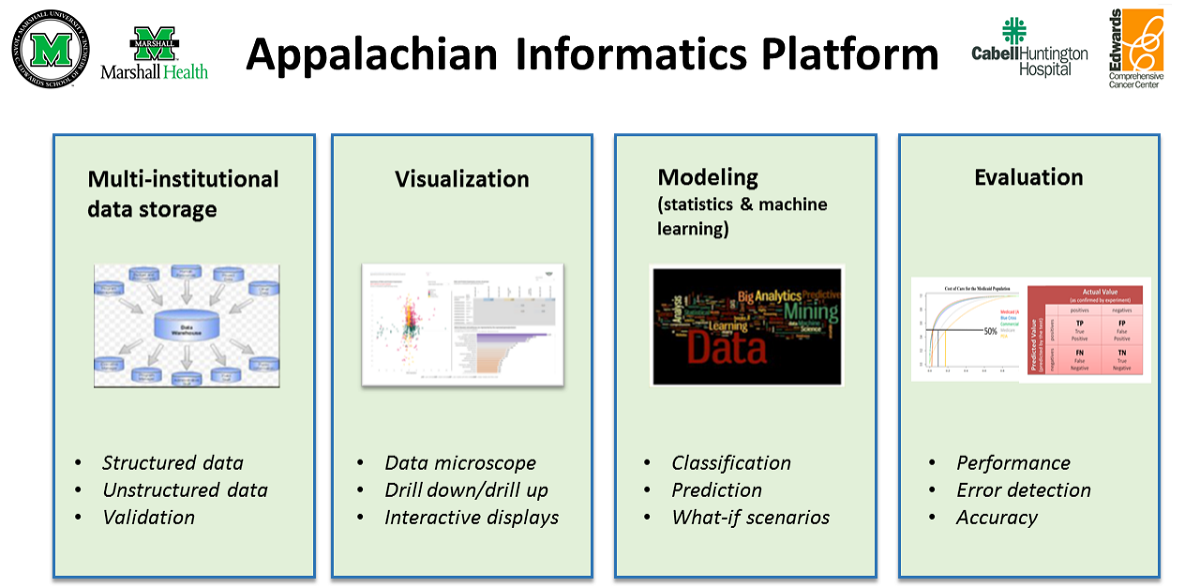
Components of the Appalachian Informatics Platform.

The ACTSI's Division of Clinical Informatics has a functional Appalachian Informatics Platform (Figure 1) that is composed of 4 major components to be described in detail in a future paper. The CDW, containing over 9 years of billing and clinical data, forms an integral part of the Appalachian Informatics Platform, which contains, in addition to the CDW, embedded data analytics (modeling and evaluation) and interactive visualization tools (eg, Tableau [Tableau Software Inc] and Power BI [Microsoft Corp]). The information contained within the CDW consists of internal structured EHR data (eg, vitals, medication, procedure, diagnosis, etc), non-EHR survey data, and unstructured (text) information received from Marshall Health practice plan, Cabell Huntington Hospital (CHH), and MU/JCESOM’s Edwards Comprehensive Cancer Center. The source data are ingested daily incrementally through SQL Server Integration Services (Microsoft Corp). These data are tested and validated via standard extract, transform, load testing, which includes but is not limited to comparing data in production systems against source data, source to target data and count testing, metadata testing, and data quality testing (accepting default values, reporting invalid data, etc). Any missing values are recoded as unknown and outliers are corrected, if possible, or recoded as unknown. The CDW serves as a secure source of quality data for research studies and development and evaluation of machine learning algorithms. It also stores the results from the resulting machine learning model following its deployment. The visual analytical tools enable initial exploratory data analysis and interactive presentation of data as well as model information for further analysis and review. After an exploratory visual analysis, detailed statistical analysis is performed using a statistics application (eg, SPSS [IBM Corp], Stata [StataCorp], R [R Foundation for Statistical Computing]).

ACTSI’s Division of Clinical Informatics accessed the ACTSI CDW to develop a data mart, called the ACTSI Maier Institute Data Mart. The ACTSI Maier Institute Data Mart comprises information about persons 65 years of age and older who have been seen at the geriatric clinic or were admitted to the primary hospital used by the school’s medical practice. It is a regional data mart specially designed to capture the unique needs of the vulnerable population in Appalachia. It supports the inclusion of socioeconomic determinants of health that greatly affect the population in this area and is supplemented by a questionnaire to gather clinical information missing from the data warehouse that is critical to dementia research. For this study, we extracted the following data for each patient aged 65 years and older between 2010 and 2019: (1) use of BZD, (2) number of BZD prescriptions, (3) dementia status, (4) visits to the CHH Emergency Department (ED), (5) source of admission and discharge disposition for inpatient admissions if available, (6) patient ZIP code, and (7) charges incurred per visit for any services received through CHH or Marshall Health physicians.

Regarding use of BZD, if a patient received one or more prescriptions of BZD or reported taking a BZD as home medication at or after the age of 65 years, they were classified as a BZD user. Otherwise, they were classified as a nonuser. A list of generic drug names used to categorize a drug as BZD are listed in Multimedia Appendix 1.

To determine the number of BZD prescriptions, the number of BZD prescriptions ordered was included, but the number of refills was not taken into account. As long as the prescription number in the EHR stayed the same, it was counted as a single prescription.

Dementia status was determined by diagnosis code. Patients that had a diagnosis code (International Classification of Diseases, 9th Revision or International Classification of Diseases, 10th Revision code) for AD at any point between 2010 and 2019 were classified as having AD, those with diagnosis codes for dementia apart from AD were classified as having other dementia, and those without any dementia diagnosis codes were classified as having no dementia. The codes are listed in Multimedia Appendix 1.

Using this extracted data, we developed an interactive dashboard that was then used for initial exploratory analysis to help outline the BZD-prescribing patterns in this population.

## Results

**Figure 2:**
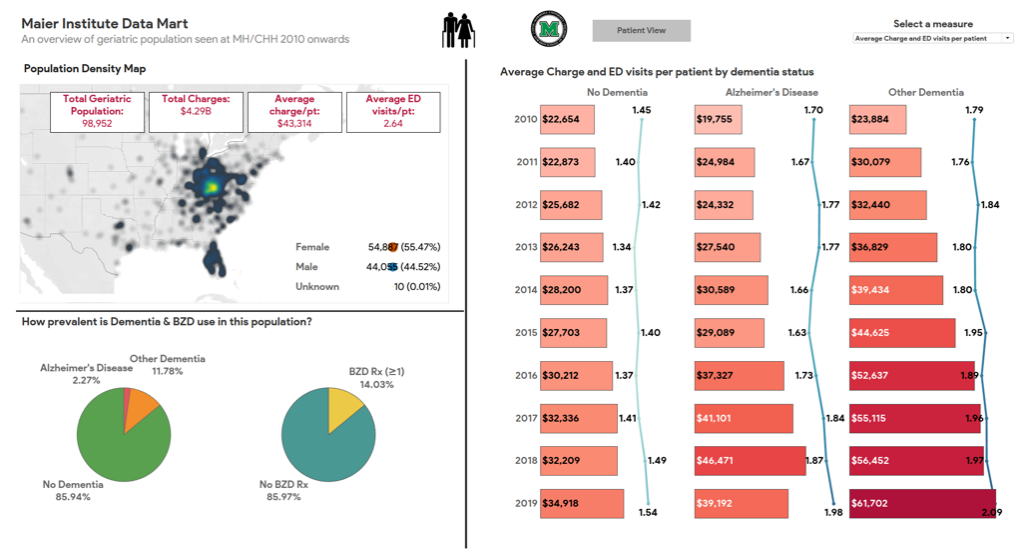
Tableau dashboard displaying key information on the geriatric population, including heat map, dementia prevalence, benzodiazepine use, and trends in charges and visits by dementia status. BZD: benzodiazepine; ED: emergency department.

Between the years of 2010 and 2019, there were 98,952 patients aged 65 and older who received any service from Marshall University physicians in CHH or the ambulatory geriatric clinic. Over the span of those 10 years, $4.29 billion in total charges were accrued, with an average charge per patient of over $43,000. The mean number of ED visits per patient was 2.64. The geriatric population was predominantly female (54,887/98,952, 55.47%) with the prevalence of dementia reaching 14.06% (13,910/98,952) (see Figure 2).

Approximately 31.24% (4346/13,910) of patients with dementia received one or more BZD prescriptions compared with 11.22% (9540/85,042) of those without dementia. A slightly higher percentage of patients specifically diagnosed with AD (761/2251, 33.81%) were found to have at least one BZD prescription. Further, fewer men than women received at least one BZD prescription (4830/44,055, 10.96% vs 9056/54,887, 16.50%) (data not shown).

**Figure 3:**
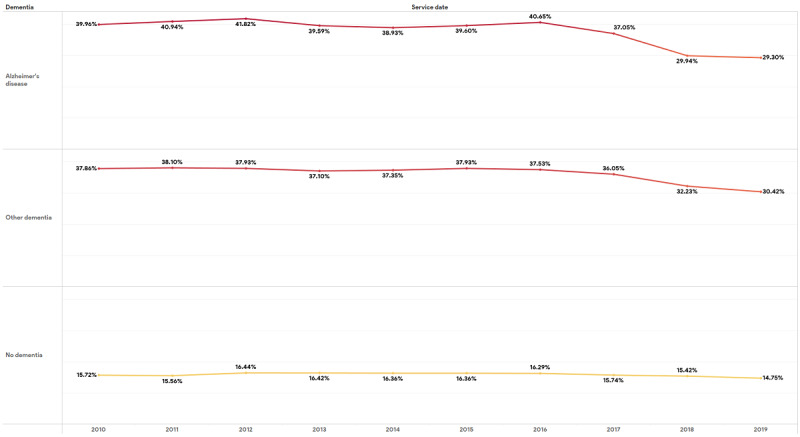
Tableau dashboard showing the trend in the percentage of patients with at least 1 benzodiazepine prescription between 2010 and 2019 by dementia status.

The percentage of elderly patients with any type of dementia receiving one or more BZD prescriptions declined appreciably (by about 10 percentage points in AD and by about 7 percentage points in other dementia from 2010 to 2019). A slight downward trend was also seen in patients with no dementia (see Figure 3).

We also found that patients with other dementia and AD, on average, incurred charges 3.3 times (approximately $109,000) and 2.3 times (approximately $76,000) those incurred by patients without dementia (approximately $33,000), respectively. The patients with any type of dementia, on average, also visited the ED 83% more compared with those without dementia (3.82 vs 2.08 visits). The average charges and number of ED visits were even higher (33%-42% increase in average charges; 34%-54% increase in average ED visits) for patients with at least one BZD prescription compared with those without a BZD prescription for patients with or without dementia.

**Figure 4:**
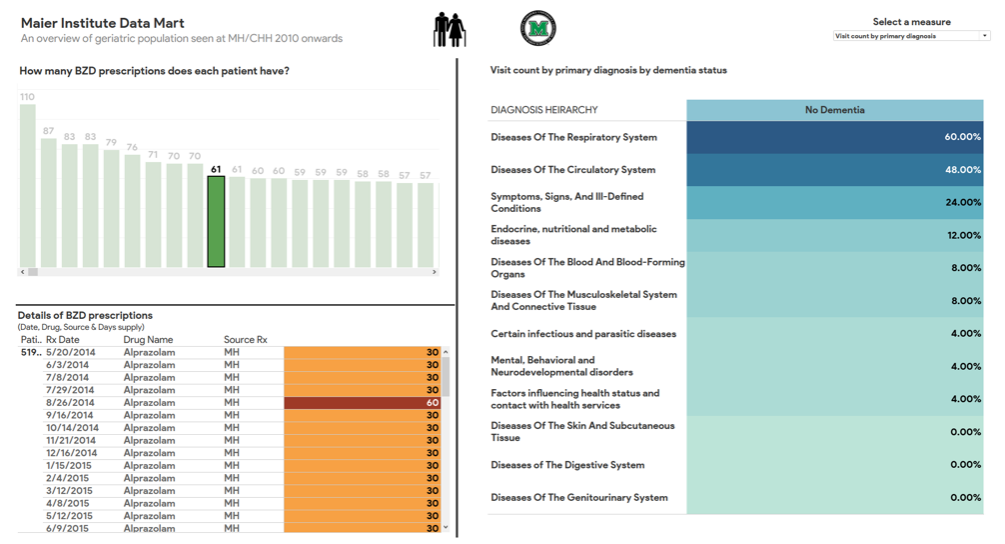
Tableau dashboard showing a patient-level view for detailed analysis (the patient IDs have been deidentified). BZD: benzodiazepine.

A patient-centered view also enabled patient-level analysis of patients’ BZD prescription history and use of health care services over the years (see Figure 4).

## Discussion

### Principal Findings and Comparison With Prior Work

The first project undertaken using the newly established ACTSI Maier Institute Data Mart explored the use of BZDs by elderly persons in our practices. Several interesting trends and patterns were noted, such as the higher prevalence of BZD use in patients with dementia and female geriatric patients and the higher mean ED visits and mean charges in patients with dementia plus at least one BZD prescription.

BZD use (receiving one or more BZD prescriptions) was found to be almost thrice as prevalent in elderly patients with dementia diagnoses compared with those without a dementia diagnosis (4346/13,910, 31.24% vs 9540/85,042, 11.22%). This is much higher than the estimate by a systematic review of past studies on BZD use in patients with AD, which estimated that 10% to 20% of these patients receive a BZD at least once during the course of the disease [27]. However, since most of the studies included in the review occurred more than 6 years ago and were heterogeneous regarding patient populations and disease stages of AD, the estimate may not accurately reflect the true prevalence of BZD use in patients with AD. Further, even though the percentage of elderly patients and patients within dementia status subgroups that received one or more BZD prescription dropped overall, the average number of prescriptions per patient rose. However, since we did not account for the number of refills per prescription and quantity of supply, it is hard to assess the implication of this finding.

Past studies have found BZD use to be more prevalent in women than in men [28,29]. This is consistent with our finding of a larger percentage of women receiving a BZD prescription compared with men.

Another study found patients with AD, in general, had more ED visits and were more likely to have a BZD-related adverse drug event, but had similar mean charges for ED visits when compared with patients without AD [30]. We found that on average, even in our population, patients with AD and other dementia visited the ED more often, but they also had higher mean charges compared with patients without dementia. Additionally, in patients receiving at least one BZD prescription over their lifetime, these numbers were even higher. This difference in charges could be because their study focused only on ED and inpatient charges, while our study included outpatient charges as well. A detailed investigation is needed to determine whether BZD use contributed to the increase in charges and ED visits.

A detailed analysis of the data is underway to better understand our initial findings, but this paper demonstrates the value of searching the data through the Appalachian Informatics Platform and exploring said data interactively with Tableau. Generally, health care providers that serve rural and indigent populations in Appalachia do not have the resources to gather and analyze quality data to understand the unique needs of the patients with dementia in this region, which as a result, remain largely unknown. In this paper, we have exemplified that these health care providers can, despite limited resources, develop and use inexpensive data warehousing and visualization tools with a small clinical informatics team to explore their data to obtain a comprehensive and near real-time picture of the current state of dementia care. This will help them identify and address care gaps, driving dementia research and quality care in the geriatric Appalachian population.

This study adds to the limited knowledge of dementia care in Appalachia through this effort. The authors hope to improve the understanding of the effect of the geographical, environmental, cultural, and socioeconomic factors on dementia care in Appalachia and in rural populations affected by similar factors through future studies.

### Limitations

We relied only on the presence of diagnosis codes specific to dementia in the billing systems to identify whether a patient had dementia. Thus, it is possible that some of the patients had dementia that was not documented in the billing system or that a dementia diagnosis was documented but later found to have been erroneous. Further, we determined BZD use based on whether a patient received a prescription for BZD or reported a BZD as a home medication. This may not indicate the actual use of BZD, since we do not know whether the patient actually filled the prescription and took the drug. Also, patients may have received BZD and other services outside our clinic or hospital. This may have resulted in an underestimation of BZD use, ED visits, and total charges.

### Future Directions: Incorporation of a Clinical Questionnaire

During this initial analysis of the ACTSI Maier Institute Data Mart, some patterns and trends emerged that warrant more detailed analysis. In the second phase of our use of the data mart, we plan to explore the clinical features of dementia. Much is known about risk factors for dementia, yet how the known risks act and interact in individual cases and whether there are other factors that contribute to the development of dementia remain unknown. Specifically, long-term clinical care of large numbers of patients with dementia by one author (SN) has raised the question of whether there could be risks specific to any of the ethnic/demographic groups in the Appalachian region of the United States or to persons with a history of exposure to environmental aspects of Appalachia.

Since the Maier Institute Data Mart is designed to serve as a repository of searchable information about these questions, we are developing a data collection instrument to gather in-depth clinical information with a more detailed patient background. This will help advance the clinical care of patients who seek evaluation and ongoing care at MU/JCESOM’s cognitive assessment clinic, known as the Susan Edwards Drake Memory Clinic. The Susan Edwards Drake Memory Clinic Questionnaire (SEDQ) will consist of 3 sections—demographics, health history, and dementia evaluation—with the resulting data integrated into the data warehouse.

In addition to commonly needed general information, which would be sought by any clinical practice, fields that will be included in the SEDQ are questions targeting the patient’s past experiences in order to gain a closer insight into specific factors that could have contributed to suspected dementia. Examples include “Where did the patient grow up? Where has the patient lived for the longest time as an adult?” “Has the patient had any past experiences with trauma or exposures?” and “Does the patient use tobacco in any form? If so, which form?”

As data accumulate in the data mart, it is expected that patterns of personal backgrounds, histories of substance usage, or toxic exposures will become apparent, and the Maier Institute Data Mart will be available for further investigation and analysis. Additionally, clinical questions that arise regarding patients of this demographic group whose information is not in the database (for a variety of reasons, for example, patients were evaluated prior to the institution of the SEDQ) can be evaluated and compared with the deidentified data of patients who are in this data mart.

The SEDQ itself will expedite the first office visit by collecting basic demographics and health history and evaluating the patient’s current mental state, as would any good previsit data tool. The SEDQ will provide even more relevant patient functionality information that is often lacking in the care of patients with dementia and their caregivers. With the responses provided on the survey, the clinician will be able to gauge the patient’s functional level through their ability to perform activities of daily living, including basic activities such as bathing and instrumental activities such as managing finances. This will improve the quality of patient care and allow the practitioner to formulate a treatment plan more efficiently. Ultimately, this process can also be examined by researchers to compare outcomes of persons evaluated at the Susan Edwards Drake Memory Clinic with the outcomes of persons evaluated and cared for elsewhere.

### Conclusions

This paper serves as a leading example of the potential ways that informatics-based research powered by the Appalachian Informatics Platform can help enhance patient care for people with dementia in Appalachia. The platform has helped improve our understanding of certain problem areas within our elderly population. We hope that it will benefit care and treatment for future patients with dementia by way of improved understanding of dementia, enhancement of existing data using data collection instruments, risk identification, care gap analysis, and comparative analysis of treatment modalities.

## Appendix

Multimedia Appendix 1 List of diagnosis codes and drug names used in the study.

## Abbreviations

ACTSI: Appalachian Clinical and Translational Science Institute
AD: Alzheimer’s disease
BZD: benzodiazepine
CDW: Clinical Data Warehouse
CHH: Cabell Huntington Hospital
ED: emergency department
EHR: electronic health record
MU/JCESOM: Marshall University Joan C Edwards School of Medicine
SEDQ: Susan Edwards Drake Memory Clinic Questionnaire
