# Supplementary material for: Analysis of Benzodiazepine Prescription Practices in Elderly Appalachians with Dementia via the Appalachian Informatics Platform: Longitudinal Study
Source: JMIR Med Inform. 2020 Aug 4;8(8):e18389. doi: 10.2196/18389 (PMC7435704; doi:10.2196/18389)
Supplement: Multimedia Appendix 1 [file medinform_v8i8e18389_app1.doc]

**Multimedia Appendix 1**

List of diagnosis codes and drug names used in the study:

1. Generic Drug Names used to categorize medication as a benzodiazepine: Alprazolam, Chlordiazepoxide, Clobazam, Clonazepam, Clorazepate, Diazepam, Estazolam, Flurazepam, Lorazepam, Midazolam, Oxazepam, Quazepam, Temazepam, Triazolam.
2. Those with the following diagnosis codes within the entire CDW were labeled as a patient with Alzheimer’s disease: ICD9 – 331.0; ICD10 – G30.0, G30.1, G30.8, G30.9.
3. Those who had diagnosis codes for other types of dementia were labeled as Other Dementia: ICD9 – 042, 046.19, 046.3, 046.8, 290.0, 290.10, 290.11, 290.12, 290.13, 290.20, 290.21, 290.3, 290.40, 290.41, 290.42, 290.43, 290.8, 290.9, 294.10, 294.11, 294.20, 294.21, 330.8, 331.11, 331.19, 331.3, 331.4, 331.5, 331.51, 331.6, 331.7, 331.82, 331.83, 331.89, 331.9, 332.0, 332.1, 333.4, 780.93, 780.97, 781.8, 797, 799.51, 799.52, 799.53, 799.55, 799.59, 907.0; ICD10 – A81.89, B20, F01.50, F01.51, F02.80, F02.81, F03.90, F03.91, G10, G20, G31.01, G31.09, G31.1, G31.2, G31.82, G31.83, G31.84, G31.85, G31.89, G31.9, R41.0, R41.1, R41.2, R41.3, R41.4, R41.81, R41.82, R41.83, R41.840, R41.841, R41.844, R41.89, R41.9, S06.9X9S
